# Anti-tumor effect of a novel PI3-kinase inhibitor, SF1126, in ^12^ V-Ha-Ras transgenic mouse glioma model

**DOI:** 10.1186/s12935-014-0105-9

**Published:** 2014-11-12

**Authors:** Alok R Singh, Shweta Joshi, Elizabeth George, Donald L Durden

**Affiliations:** UCSD Department of Pediatrics, Moores UCSD Cancer Center, University of California School of Medicine, San Diego, CA 92093 USA; Emory University School of Medicine, Atlanta, GA USA; Division of Pediatric Hematology-Oncology, UCSD Rady Children’s Hospital, La Jolla, CA USA

**Keywords:** ^12^ V-Ha-Ras-astrocytoma cells, SF1126, Proliferation, EGF, α_v_β_3_ integrin, Migration, Xenograft model

## Abstract

**Background:**

Growth factor mediated activation of RAS-MAP-kinase and PI3-kinase-AKT pathways are critical for the pathogenesis of glioblastoma. The attenuation of PI3-kinase/AKT signaling will be effective in regulating the tumorigenic phenotypes of the glioma cells.

**Methods:**

Glioma cells derived from the brain of the ^12^ V-Ha-Ras transgenic mice were used to study the effect of PI-3 kinase inhibitor SF1126 on activation of AKT and ERK signaling, proliferation, vitronectin mediated migration and changes in the distribution of cortical actin on vitronectin in the glioma cells in vitro. The anti-tumor effects of SF1126 were also tested in vivo using pre-established tumors (subcutaneous injection of the glioma cells from ^12^ V-Ha-Ras transgenic mice) in a mouse xenograft model.

**Results:**

Our results demonstrate that treatment of LacZ+, GFAP + and PCNA + ^12^ V-Ras Tg transformed astrocytes with SF1126 and LY294002 blocked the activation of AKT as well as EGF-induced phospho-ERK. Most notably, treatment of SF1126 blocked integrin-dependent migration in transwell and scratch assays and caused a significant change in the organization and distribution of cortical actin on vitronectin in the glioma cells. Moreover, SF1126 treatment inhibited in vitro proliferation of these cells and in vivo growth of pre-established subcutaneous tumors in a xenograft model.

**Conclusion:**

The present study validate the potent anti-proliferative and anti-migratory activity of SF1126, in a V^12^ Ras oncogene driven glioma model and suggest that this effect is mediated potentially through a combined attenuation of PI3-kinase and MAP-kinase signaling pathways.

## Introduction

Glioblastoma is the most common and lethal type of adult brain tumor [1,2]. Aberrant growth factor receptor-mediated signals and oncogenic alterations of tumor-associated genes are the hallmarks of prognostically poor and aggressive gliomas. One of the most frequently encountered genetic events occurring in high-grade gliomas is the overexpression/amplification/gain-of-function mutation of epidermal growth factor receptor (EGFRvIII) gene [3-5]. However, results from various studies using different transgenic mouse models of human glioma (GFAP-^12^ V-Ha-Ras-transgenic mice or GFAP-EGFRvIII; GFAP-V^12^Ha-Ras transgenic mice indicate that the expression of astrocyte-specific EGFRvIII (mutated receptor) alone is not sufficient for the gliomagenesis [6-10]. Studies conducted by Guha et. al., showed that an upregulated EGFR signal (from EGFRvIII) co-operates with the activated p21-RAS pathway (elevated levels of activated p21-RAS) towards the pathogenesis and molecular progression of the malignant gliomas [6-10]. Upregulation of the RAS pathway and activation of RAS-effectors have been reported in glioblastoma multiforme (GBM) [11] and a functionally relevant activation of the p21-RAS pathway has been reported to be associated with the pathogenesis and the progression of the disease (despite the lack of oncogenic mutations of p21-RAS in glioblastoma) [6,9,11].

Another most common genomic alteration that occurs in GBM in addition to EGFR mutations is the deletion, loss of expression and/or mutation of PTEN tumor suppressor gene [12-15]. The fact that, (i) PTEN is frequently mutated or lost in GBM (frequency of ~70–90%), and (ii) PTEN status is of prognostic importance in GBM patients indicates the important role of PTEN/PI3-kinase pathway in the progression and the outcome of the disease. Several studies have shown that signals arising out of oncogenic alterations in glioblastomas are mediated through the PI3-kinase/AKT pathway [14,16]. Recent studies have also reported genetic changes in different catalytic subunits of PI3-kinase in glioblastomas [14,17]. Furthermore, several studies (using tumor samples and tumor-derived cell lines) have indicated that PI3-kinase-AKT dependent pathways contribute to the malignant phenotypes like survival, migration and tumor induced angiogenesis in GBM [18,19].

Considering the reports that growth factor mediated activation of RAS pathway causes an activation of PI3-kinase [20], and that a combined activation of RAS-MAP-kinase and PI3-kinase-AKT pathways are critical for the pathogenesis of glioblastoma [21], we hypothesized that the attenuation of PI3-kinase/AKT signaling will be effective in regulating the tumorigenic phenotypes (proliferation and integrin-mediated migration) of the glioma cells derived from GFAP-^12^ V-Ha-Ras transgenic mice. Our previous studies have well-established the anti-tumor and anti-angiogenic activity of SF1126 [22-24]. Herein we provide evidence for the anti-tumor effects of SF1126, in a transgenic mouse model for human glioma. We show that the blockade of the PI3-kinase pathway alone is effective for the inhibition of growth and integrin-dependent migration of these cells in vitro and in vivo.

## Materials and methods

### Cell lines and reagents

U87MG glioma cell line was purchased from the American Tissue Culture Collection (Rockville, MD). These cell lines were propagated in RPMI (Invitrogen, Carlsbad, CA), with 10% FBS (Hyclone) and 1% penicillin/streptomycin as described before [25]. Antibodies against phospho-AKT (ser473), AKT, PTEN, phospho-ERK (p42/44 MAP kinase; Thr202/Tyr204), ERK (p42/44 MAP kinase) and PCNA were purchased from Cell Signaling Technology (Beverly, MA). Tubulin and anti-CD31 antibodies were procured from BD Biosciences (San Jose, CA). Anti-GFAP antibody, rabbit ImmunoCruz Staining system, mouse ImmunoCruz Staining system and rat ABC staining system were procured from Santa Cruz Biotechnology, Inc, (Santa Cruz, CA). LY294002 and PD98059 were purchased from Calbiochem (San Diego, CA). Vitronectin, β-actin and X-gal were purchased from Sigma-Aldrich, St. Louis, MO. RGDS was procured from Biomol International (Plymouth, PA). HRP-tagged anti-rabbit IgG and anti-mouse IgG were obtained from Amersham Life Sciences (UK). Goat anti-mouse and anti-rabbit IgG (H + L)-AP (human adsorbed) were purchased from Southern Biotechnology, Inc. (Birmingham, Alabama). SF1126 is a vascular targeted pan PI-3 kinase drug developed in collaboration with SignalRx pharmaceuticals.

#### Animal studies

^12^ V-Ha-Ras transgenic mice were obtained from Dr. Guha (Washington State University, St. Louis, Missouri) and maintained according to an IACUC-approved protocol in the Animal Facility Core at Emory University. Ninety-five percent of these ^12^ V-Ha-Ras transgenic mice are reported to die from solitary or multifocal low- and high-grade astrocytomas within 2–6 months [6]. These transgenic astrocytomas are reported to be pathologically similar to human astrocytomas, with a high mitotic index, nuclear pleomorphism, infiltration, necrosis, and increased vascularity. Expression of the transgene in ^12^ V-Ha-Ras mice (tail biopsy samples of the mice from which the derivative glioma cells cultures were established) and in the derivative glioma cells (established from the transgene-expressing mice) at different passages (8^th^, 70^th^, and 100^th^ passages) were confirmed by genotyping as described by Guha et al. [6,8]. Athymic female mice (CD-1 *nu/nu*, 20–25 grams) were obtained from the NIH/NCI repository. In vivo studies were carried out according to the protocol (care and use of animals for experimental purpose) that has been approved by the Animal Facility Core at Emory University.

### Derivation of astrocytoma cell lines

Brain astrocyte cultures were initiated from wild type (WT) mice (1-day-old neonates) and ^12^ V-Ha-Ras transgenic mice (3–4 months old animals). ICR strain of mice was used as WT mice. Brain tissue was dissected (on ice) and was subjected to trypsin (0.25%) digestion for 5–7 minutes at 37°C. Digested tissue was triturated in presence of DNase I (Invitrogen, Carlsbad, CA). The resulting cells were grown in DMEM-F12 (Invitrogen, Carlsbad, CA) with 10% FBS, 1% penicillin and streptomycin at 37°C in a humidified atmosphere of 5% CO_2_ in air. These cells were used for analysis of astrocytic marker GFAP by immunohistochemistry (>95% GFAP positive at passage 8). Cultures of astrocytic tumor cell lines from ^12^ V-Ha-Ras transgenic mice were continued to the successive passages (currently at passage 100). Primary astrocytes were lost at passage 4–5.

### LacZ staining

Expression of the transgene in astrocytic tumor cell lines derived from ^12^ V-Ha-Ras mice (8^th^, 70^th^, and 100^th^ passages) was confirmed by the histochemical staining for LacZ activity. In brief, fixed cells were incubated in X-gal (5%) solution at 37 °C in a CO_2_ incubator for 6–10 hours. U87MG glioma cell line was used as the negative control.

### Immunohistochemistry

U87MG glioma cells and astrocytoma cells from ^12^ V-Ha-Ras mice (8^th^, 70^th^, and 100^th^ passages) were immunohistochemically stained for GFAP (H-50; 1: 50 rabbit polyclonal antibody) and PCNA (1: 2000 mouse monoclonal antibody). Immuno-staining for secondary antibodies was carried out using rabbit ImmunoCruz Staining system and mouse ImmunoCruz Staining system respectively.

### PI3-kinase activity assay

PI3-kinase activity was determined by measuring the amount of ATP consumed (remaining in the reaction mixture) following a kinase reaction (for 30 minutes) using Kinase-Glo Luminescent assay kit (Promega Corporation, Madison WI). Kinase reactions were carried out in 96 well assay plates containing 50 μl of kinase buffer (40 mM Tris, 20 mM MgCl_2_, 0.1 mg/ml BSA; pH 7.5) 1.0 μM ATP, 5 μM PIP_2_ (substrate) and 1 μg of recombinant p110α PI3-kinase enzyme (Upstate Biotechnology) in the presence or absence of 10 μM of the inhibitors (SF1126 or LY294002). Luminescence was read in an Envision 2102 (Perkin-Elmer Life and analytical Sciences) multi-label counter following the addition (10 minutes) of the Kinase-Glo reagent (50 μl). Data points represent the average of triplicate readings.

### Biochemical analyses

For all Western blots, 2 × 10^6^ cells were plated in 10 cm tissue culture dishes such that the density of the cells at the time of lysis was 70–80% confluent. Cells were allowed to adhere overnight and next day were treated with LY294002 (25 μM and 50 μM) or PD98059 (20 μM) or RGDS (50 μM pre-pulse for 30 minutes). SF1126 was diluted in PBS containing 10% FBS. Whole cell lysates were prepared using RIPA buffer (50 mM Tris–HCl, pH 7.6, 150 mM NaCl, 100 mM NaF, 1 mM EDTA, 1 mM EGTA, 0.05% NP40, 1% aprotinin, 0.01 mg/ml leupeptin and 0.08 mM PMSF). Equivalent amounts of protein (30–50 μg/lane; Bradford assay) were resolved in 10% SDS-PAGE and transferred to nitrocellulose membrane as described before [26]. Membranes were probed with anti-sera specific for PTEN, AKT, phospho-S473-AKT, Phospho-ERK, ERK, tubulin and β-actin. Individual bands were visualized by chemiluminescence reagent ECL (Amersham Pharmacia biotech, UK).

### Integrin-induced migration assays

Haptotaxis and wound healing assays were performed to test the integrin-directed migration of the cells. Haptotaxis was carried out using transwell migration chambers (Costar Corp., Cambridge, MA) as previously described [27]. In brief, cells (2 × 10^5^/well) were added on top of the membrane (of the upper chamber of the transwell) containing 8 μm pore through which they were allowed to migrate over 24 hours to the vitronectin-coated (10 μg/ml for 1 hour) side. In vitro wound healing migration assays were performed (scratch wound model) as described previously [28,29]. In brief, wounds were created by scratching the confluent monolayer of cells adhered on vitronectin (10 μg/ml) coated plates. Migrated cells (24 hours) were stained (crystal violet), photographed, and counted from randomly chosen fields using Olympus DP70 system. Student’s *t*-test was used to determine the statistical significance.

### Actin dynamics

Derivative glioma cells plated on vitronectin-coated cover slips were treated with 50 μM SF1126 for 30 minutes and were then processed for Phalloidin-555 staining of filamentous actin. Nuclei were counter stained with DAPI. Stained cells were photomicrographed using a Zeiss (Thornwood, NY) LSM 510 Meta confocal microscope with a 63x (1.4-numerical-aperture) or 100x (1.4-numerical-aperture) Plan-Apochromat oil objective. Images were acquired using Zeiss LSM 510 software and processed in Adobe Photoshop 7.0 as described before [30].

### Time-lapse video imaging of live cell

A scratch assay (wound-healing Assay) was performed on the confluent monolayer of the derivative glioma cells plated on vitronectin coated glass bottom culture dishes (MatTek Corp., Ashland, MA). The plates were placed in the live-cell imaging chambers (equipped with a 37°C stage warmer, incubator, and humidified 5% CO_2_ perfusion). Digitized bright-field time-lapse images of the movement of the cells into the scratched area in presence or absence of SF1126 (50 μM) were acquired with a Perkin Elmer Ultraview ERS (Norwalk, CT) disk-spinning confocal system, mounted on a Zeiss Axiovert 200 M inverted microscope. Images of multiple optical slices were collected (at 2×2 binning) for 496 minutes (approximately 8 hours) with a Hamamatsu Orca ER camera (Middlesex. NJ) using a Plan-Neofluar 40× phase objective (NA 0.75) at 5 minutes interval for each image set. To account for the axial focal changes of cells as they move, 16 optical sections were collected at 0.95 μm interval spacing with the Perkin-Elmer Ultraview ERS spinning disk confocal system fitted to a Zeiss Axiovert 200 M inverted microscope that was enclosed within the temperature controlled chamber as described [31]. Once acquired, images were exported in TIF format and imported into Metamorph 6.1 (Universal Imaging, Downingtown, PA). At the end of 8 hours, cells were allowed to move for a total of 24 hours, stained with Phalloidin-555 for actin polymerization. Migration of cells from the boundary of the scratch was quantified by establishing the trajectory of each nucleus and measured by tracking the central nuclear area of those cells starting at the scratch border using the “Track Points” feature of the program. Each trajectory is then characterized by two quantitative motility descriptors, namely the “average velocity” and the “maximum relative distance from the origin” (the MRDO variable) of each cell using Metamorph 6.1 (Universal Imaging, Downingtown, PA). The MRDO variable is the greatest linear distance between a cell’s original position and its final position normalized by the observation time for the cell analyzed.

### Tumor xenograft experiments

Athymic female mice (CD-1 *nu/nu*, 20–25 grams) were used for in vivo tumor growth inhibition studies. Five million astrocytoma cells (in 100 μl PBS) derived from ^12^ V-Ha-Ras transgenic mice were injected subcutaneously into the right flank of each mouse. Tumor growth was monitored twice per week for external measurements using Vernier calipers. Tumor volume was calculated using the formula V = (A × B ^2^)/2 where A and B represent length and width of the tumor respectively. For SF1126 experiments, treatment was initiated when tumors reached a tumor volume of 100 mm^3^. Mice were divided randomly into 2 groups receiving vehicle (acidified sterile water) or SF1126 (50 mg/kg, subcutaneous) 3 times weekly (Monday, Wednesday and Friday) for 3 weeks as described before [22]. Animals were monitored for the signs of clinical toxicity and changes in body weight.

### CD31 and PCNA immunohistochemistry in the tumor tissues

At the end of the efficacy studies, tumors were harvested and placed in OCT blocks for frozen section analysis or fixed in 10% buffered formalin and/or processed into paraffin. Sections of tumor tissue at 4 μm thickness were stained with rat anti-mouse CD31 antibody for detection of the murine tumor microvasculature. Quantitation of microvessel density (MVD) was performed as described before [27]. PCNA staining was done using paraffin sections of the tumor tissues (1: 2000 mouse monoclonal antibody). Immuno-staining for secondary antibodies was carried out using rabbit ImmunoCruz Staining system and mouse ImmunoCruz Staining system.

### Statistical analysis

Student’s *t*-test was used to evaluate differences observed between the experimental groups and to compare tumor volume differences between SF1126 treated mice and the vehicle treated controls.

## Results

### Expression of ^12^ V-Ha-Ras transgene in astrocytoma cells from ^12^ V-Ha-Ras transgenic mice

**Figure 1:**
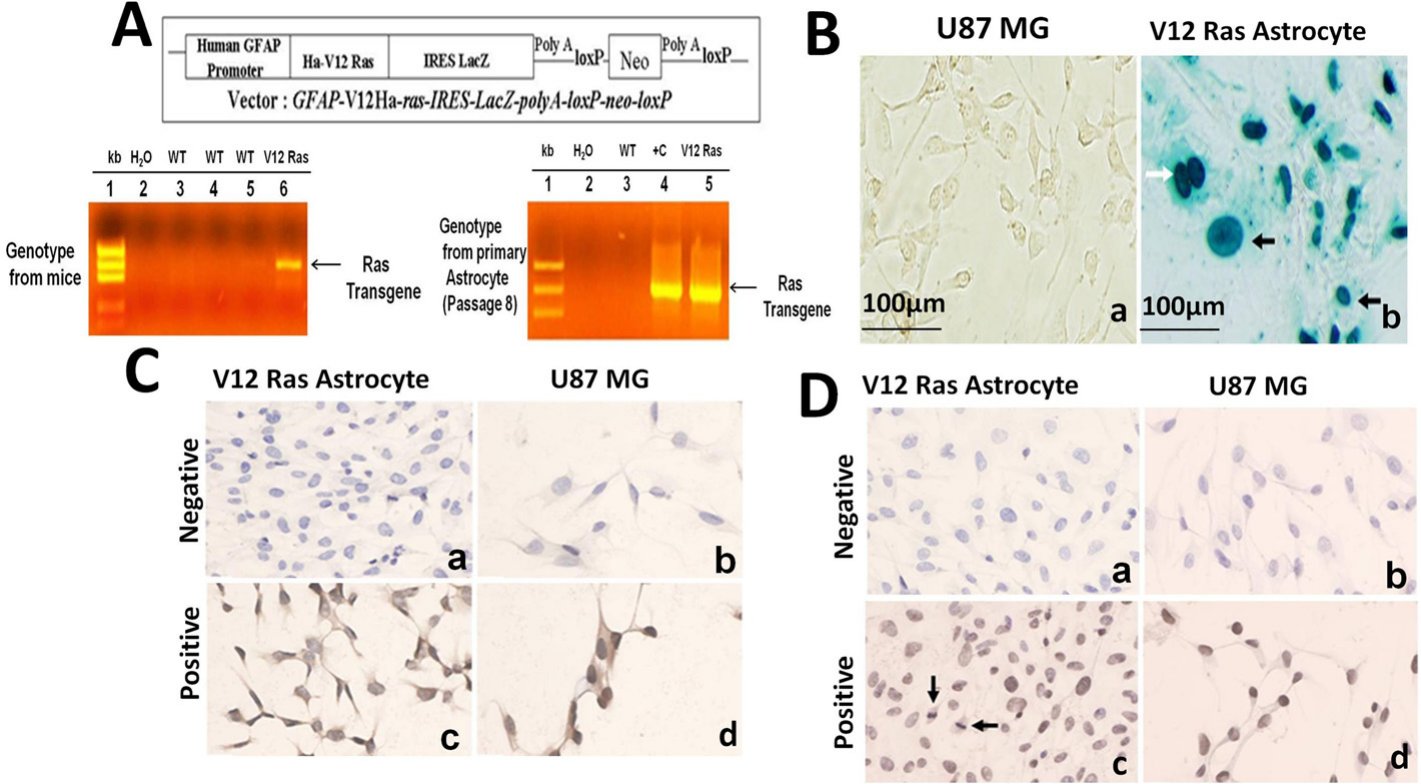
**Immuno-histochemical characterization of the astrocytes derived from** ^**12**^ **V-Ha-Ras transgenic mice. A**. Upper panel shows schematic representation of the vector for the transgenic mice. Lower panel shows the presence of transgene in ^12^ V-Ha-Ras transgenic mice (left panel) and primary astrocytes (passage 8) derived from the V^12^ Ras transgenic mice (right panel) as confirmed by genotyping. Lane 2 and 4 n right panel shows negative and positive control respectively. **B**. Histochemical staining for LacZ in the astrocytes derived from ^12^ V-Ha-Ras transgenic mice. Derivative astrocytes from ^12^ V-Ha-Ras transgenic mice (passage 8) were stained for LacZ as a transgene marker using X-gal. Cells show nuclear positivity for LacZ (photomicrograph b). LacZ stained nuclei show prominent nuclear atypia (black arrowheads in the photomicrograph b) and dividing nucleus (white arrowhead in the photomicrograph b). U87MG glioma cells served as negative control (photomicrograph a). **C & D**. Immunostaining for GFAP (C) and PCNA (D) in the derivative astrocytes from ^12^ V-Ha-Ras transgenic mice as described in Materials and methods. Derivative astrocytes cells show cytoplasmic positivity for GFAP or nuclear positivity for PCNA (photomicrograph c) in **C & D**. U87MG glioma cells are used as negative control (stained without primary antibody) and positive control (stained with primary antibody) as shown in photomicrograph b and photomicrograph d respectively.

In order to confirm the presence of transgene in the transgenic mouse as well as in the astrocytoma cells derived from the brain of the transgenic mouse, genotyping was performed. Upper panel of Figure 1A illustrates the schematic representation of the vector used for the generation of transgenic mice. The left bottom panel (lane 6) and right bottom panel (lane 5) of Figure 1A shows the presence of the transgene in the mouse (and the astrocytoma cells derived from that mouse. Once the astrocytoma cell lines were established, the genotype was repeated at different passages (8^th^, 70^th^, and 100^th^) to verify the presence of the transgene.

In order to further confirm the presence of the transgene in glioma cells from ^12^ V-Ha-Ras transgenic mice, we tested the LacZ activity in these cells at different passages (8^th^, 70^th^, and 100^th^). It is already reported that the *IRES* fragment of the *GFAP* promoter-driven transgene construct (*GFAP*-V12Ha-*ras*-*IRESLacZpolyA-loxP-neo-loxP)* allows independent translation of LacZ [6]. Figure 1B shows the LacZ (+) cells in the monolayer of glioma cells isolated from GFAP V12 Ras mice (photomicrograph b) as compared to the negative control (U87MG glioma cells; photomicrograph a). Shannon et al. [6,8] have reported similar LacZ (+) nuclear staining of astroglial cells in ^12^ V-Ha-Ras transgenic mice (from E16.5). Interestingly, these LacZ (+) V^12^-Ras-astrocytes showed distinct nuclear atypia, which characterizes the transition to neoplasia as described by Shannon et al. [6,8].

The *GFAP* promoter-driven (*GFAP*-V12Ha-*ras*-*IRESLacZpolyA-loxP-neo-loxP)* transgene construct expresses one mRNA that encodes for both ^12^ V-Ha-Ras and LacZ proteins [6]. In order to test the presence of this specific astrocytic marker in the glioma cells from ^12^ V-Ha-Ras transgenic mice, we performed immunohistochemistry (IHC) for GFAP at different passages (8^th^, 70^th^, and 100^th^). Figure 1C shows the GFAP (+) V^12^-Ras-astrocytes (photomicrograph c) showing the expression of GFAP in these cells. U87MG glioma cells were used as positive control (photomicrograph d, Figure 1C). V^12^-Ras-glioma cells (photomicrograph a, Figure 1C) and U87MG glioma cells (photomicrograph b, Figure 1C) not stained with primary antibody were used as negative control. Consistent with our results, Shannon et al., have also reported similar GFAP positivity of astroglial cells in 3 weeks old ^12^ V-Ha-Ras transgenic mice [6,8]. In order to explore the proliferation of astrocytes derived from ^12^ V-Ha-Ras transgenic mice we performed IHC for the proliferating cell nuclear antigen (PCNA). Figure 1D showed that PCNA primarily stained the GFAP (+) V^12^-Ras-astrocytes (photomicrograph c). U87MG glioma cells were used as positive control (photomicrograph d, Figure 1D). V^12^-Ras-glioma cells (photomicrograph a, Figure 1D) and U87MG glioma cells (photomicrograph b, Figure 1D) not stained with primary antibody were used as negative control.

### SF1126 and LY294002 inhibit PI3-kinase activity

**Figure 2:**
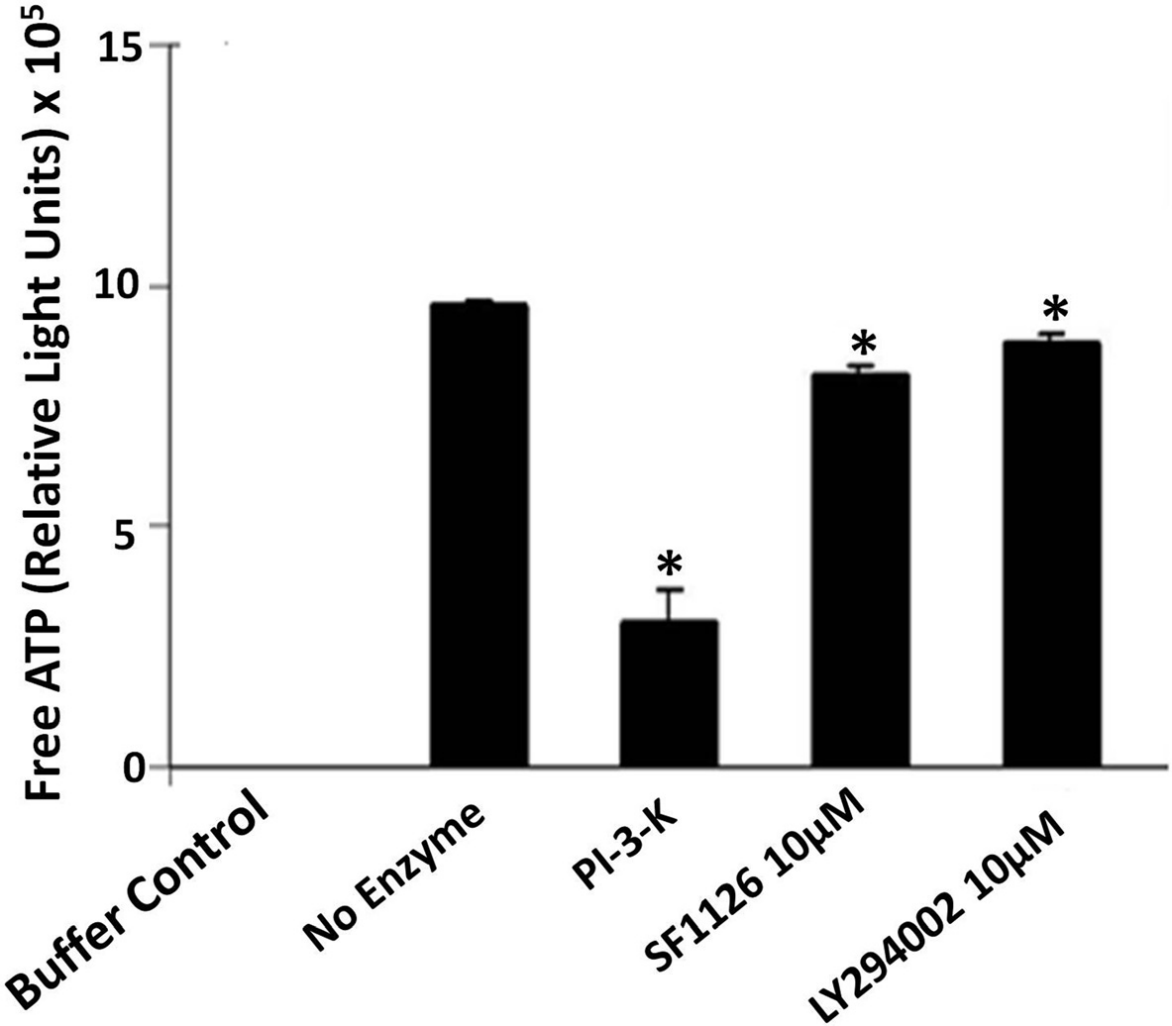
**SF1126 and LY294002 inhibit in vitro PI3-kinase activity in cell free system.** Effects of SF1126 and LY294002 on the enzymatic activity of PI3-kinase was determined in a cell free system by measuring the amount of unused ATP (free ATP remaining in the reaction mixture) following the kinase reaction (for 30 minutes) using Kinase-Glo Luminescent assay kit according to the manufacturer’s protocol. Bars are the mean ± SD of relative light unit (RLU) readings (in triplicates) representing of the amount of free ATP in the respective reaction mixtures. *P < 0.05. Experiment was repeated thrice.

The results mentioned in Figure 1 clearly depict that ^12^ V-Ha-Ras transgene is expressed in astrocytoma cells derived from ^12^ V-Ha-Ras transgenic mice. We next examined the ability of PI-3 K inhibitors, LY294002 and SF1126 to block in vitro kinase activity of PI3-kinase in GFAP (+) V12-Ras-astrocytes. Figure 2 shows that 10 μM of SF1126 or LY294002 significantly inhibited the PI3-kinase activity in vitro (in cell free system) in these cells compared to the recombinant PI3-kinase enzyme activity (1 μg) in presence of 5 μM PIP_2_ substrate.

### Effects of SF1126 and LY294002 on the levels of phospho-AKT in glioma cells from ^12^ V-Ha-Ras transgenic mice

**Figure 3:**
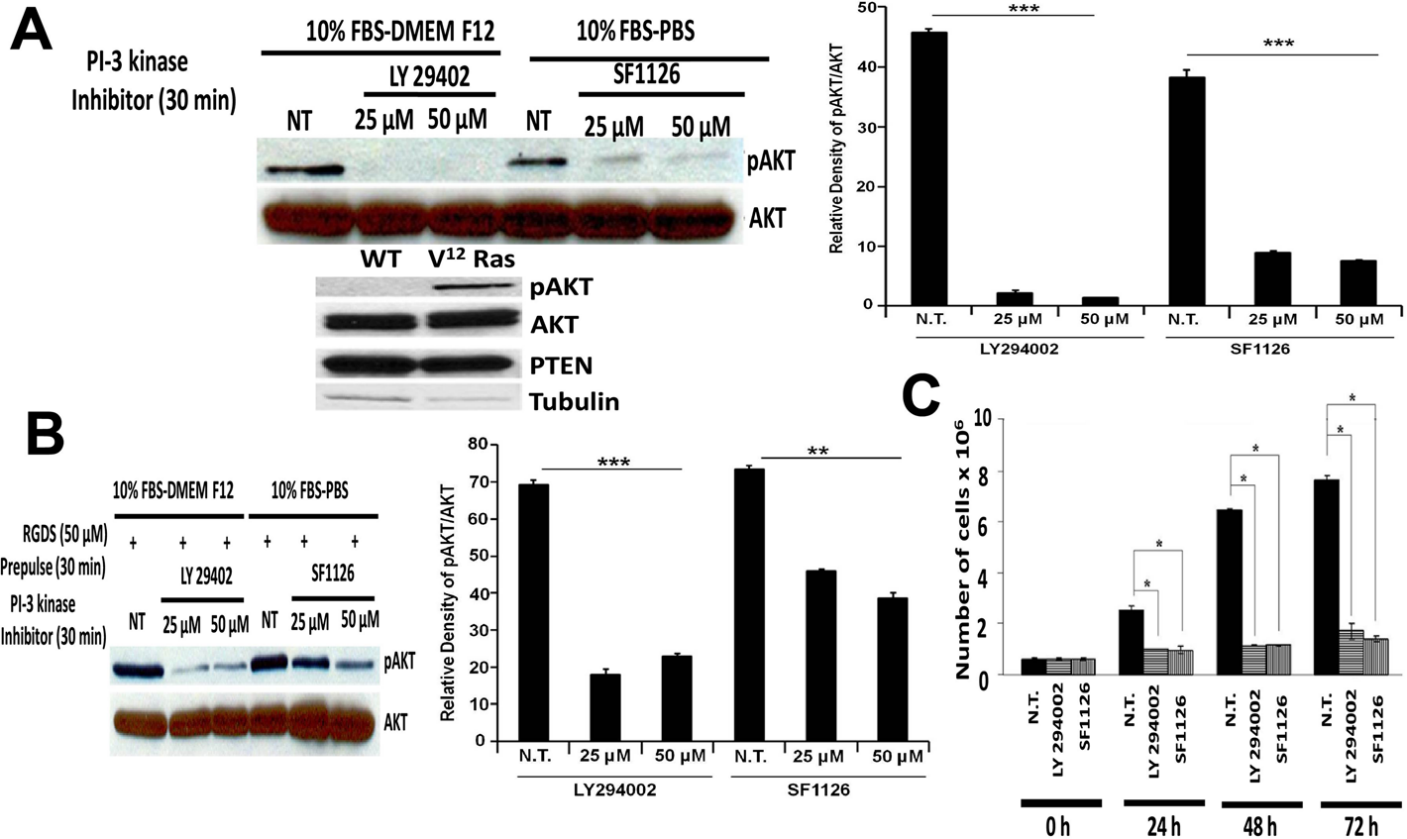
**Administration of SF1126 and LY294002 blocked the activation of AKT and inhibited the in vitro growth of glioma cells from** ^**12**^ **V-Ha-Ras transgenic mice. A**. Left panel shows that treatment of SF1126 and LY294002 (25 μM and 50 μM) significantly decreased the levels of phospho-AKT. Right panel shows the densitometry analysis of pAKT band relative to AKT. Bottom panel shows endogenous levels of phospho-AKT and PTEN in the derivative glioma cells from the ^12^ V-Ha-Ras transgenic mice as compared to the primary astrocytes from the wild type animal. **B**. Left panel shows the effect of pre-pulse of RGDS peptide on the inhibition of levels of phospho-AKT following the treatment of SF1126 and LY294002 in glioma cells from the ^12^ V-Ha-Ras transgenic mice. Cells were pulsed with RGDS peptide (50 μM) for 30 minutes prior to the treatment of SF1126 (25 μM) or LY294002 (25 μM). Phospho-AKT levels were determined 30 minutes after the treatment of the inhibitors. Right panel shows the densitometry analysis of pAKT band relative to AKT. **C**. Effects of SF1126 and LY294002 on the in vitro growth of the derivative glioma cells from the ^12^ V-Ha-Ras transgenic mice. Derivative glioma cells were treated (50 μM) with SF1126 or LY294002 and the growth of the cells were determined from the cell counts (trypan blue) 24, 48 and 72 hours following the administration of the inhibitors. Bars represent the mean ± SD of readings from 4 independent experiments. *P < 0.05. Experiments were repeated three times with similar results.

We next explored the effect of SF1126 and LY294002 on the PI3-kinase/AKT pathway in glioma cells isolated from ^12^ V-Ha-Ras transgenic mice. Figure 3A (Left panel) shows that the treatment of 25 μM and 50 μM of SF1126 or LY294002 for 30 minutes completely abrogated the levels of phospho-AKT. Figure 3A (Right panel) depicts the densitometry analysis of Western blot showing relative quantification of pAKT. It is well documented that the status of phospho-AKT in a cell is dependent on the levels of endogenous PTEN so we next determined the endogenous levels of PTEN in these derivative glioma cells compared to the astrocytes from the wild type (WT) animals (Figure 3A, Bottom panel). Figure shows that V^12^-Ras-derived glioma cells contain higher baseline levels phospho-AKT than the WT astrocytes despite comparable levels of endogenous PTEN. Interestingly, this might indicate that the levels of phospho-AKT in V^12^-Ras-astrocytoma cells may be additionally controlled by the status of RAS activation in these cells, independent of the levels of endogenous PTEN. This result further explains the higher rate of proliferation of V^12^-Ras-astrocytoma cells as compared to the WT astrocytes (data not shown).

### Effects of pre-pulse of RGDS peptide on the levels of phospho-AKT following the treatment of SF1126 or LY294002 in glioma cells from ^12^ V-Ha-Ras transgenic mice

SF1126 is a RGDS-conjugated pro-drug. We argue that if the effect of SF1126 in a cell is mediated through RGDS or RGDS binding and internalization of the conjugate, then a pre-pulse of RGDS peptide will specifically block the effect of SF1126 on the levels of phospho-AKT. On the contrary, a similar pre-pulse of RGDS peptide will not affect the action of LY294002 in these cells. Hence, we tested the effect of pre-pulsing of RGDS peptide on the effect of SF1126 and LY294002. Figure 3B (Left panel) shows that pre-pulse of RGDS (50 μM for 30 minutes) blocked the inhibitory effect of SF1126 (at 25 μM and 50 μM concentration) on the levels of phospho-AKT without affecting the inhibitory effect of LY294002 (at similar concentration). Figure 3B (Right panel) depicts the densitometry analysis of Western blot showing relative quantification of pAKT.

### Effects of SF1126 and LY294002 on proliferation of glioma cells from ^12^ V-Ha-Ras transgenic mice in vitro

The results mentioned in Figure 3A clearly depict higher levels of endogenous phospho-AKT in V^12^-Ras-astrocytes from ^12^ V-Ha-Ras transgenic mouse than the primary astrocytes from wild type (WT) mouse. However, a significant decrease in the levels of phospho-AKT following SF1126 treatment in derivative V^12^-Ras-astrocytes (Figure 3A and B) indicated that SF1126 can attenuate the PI3-kinase pathway in these cells. Fan et al., has reported that inhibition of cell proliferation in glioblastoma is mediated via the downregulation of PI3-kinase/AKT signaling pathway [16]. Hence we next studied the effect of SF1126 on proliferation of glioma cells from ^12^ V-Ha-Ras transgenic mice in vitro and compared it with the effect of LY294002. Figure 3C shows that the treatment (50 μM) of SF1126 and LY294002 significantly inhibited cell growth over a period of 72 hours in vitro. This represents the first direct evidence that SF1126 can function as potent PI-3 kinase inhibitor in glioma cells.

### Effects of SF1126 on the baseline levels of phospho-ERK in glioma cells from ^12^ V-Ha-Ras transgenic mice

**Figure 4:**
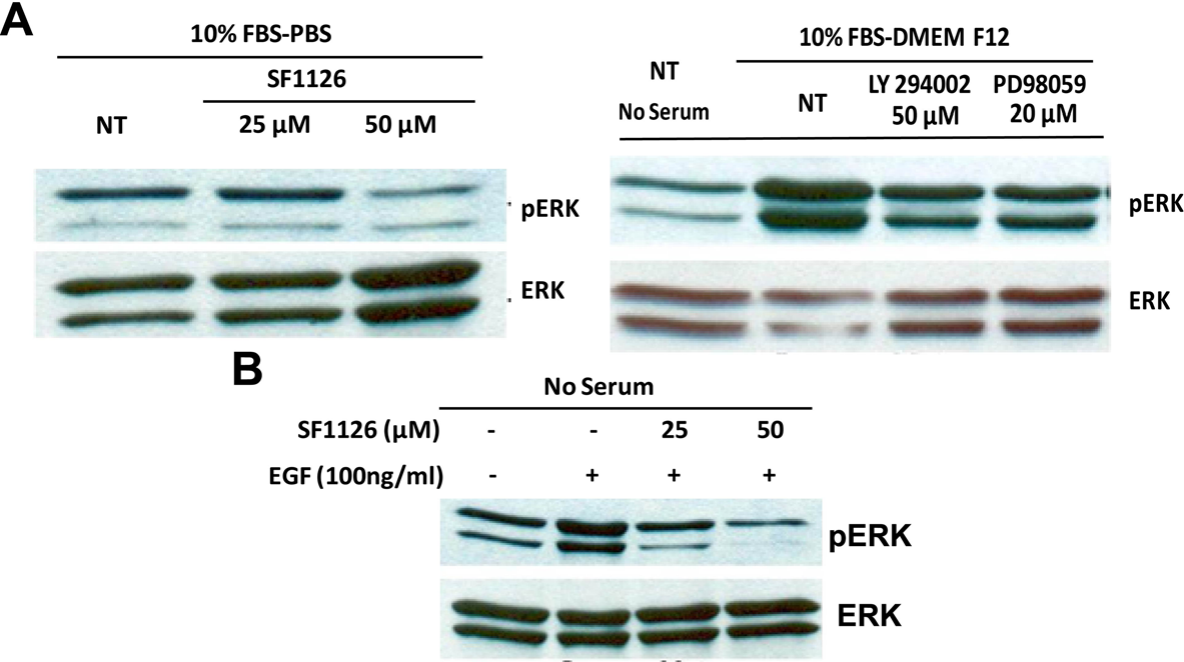
**Administration of SF1126 decreased the baseline levels of phospho-ERK and EGF-induced activation of ERK in the glioma cells from** ^**12**^ **V-Ha-Ras transgenic mice. A**. Left panel shows effect of SF1126 on the levels of phospho-ERK in the glioma cells from ^12^ V-Ha-Ras transgenic mice. Derivative glioma cells grown in 10% serum conditions were treated with two different concentrations (25 μM and 50 μM) of SF1126. Right panel shows that treatment of LY294002 (50 μM) and PD98059 (20 μM) blocked the activation of ERK in the derivative glioma cells. **B**. Effect of SF1126 on EGF-stimulated levels of phospho-ERK in the derivative glioma cells from ^12^ V-Ha-Ras transgenic mice. Glioma cells cultured in serum free media were stimulated with EGF (100 ng/ml) and treated with 25 μM and 50 μM of SF1126. Experiments were repeated three times with similar results.

It is well established that RAS/MAP kinase/ERK signaling plays an important role in the proliferation of tumor cells in gliomas and upstream activation of RAS leads to the downstream activation of PI3-kinase [20,32-34]. So, we next examined the ability of SF1126 to inhibit the baseline levels of phospho-ERK in these glioma cells and compared this effect with LY294002 and a selective MEK inhibitor, PD98059. Left panel in Figure 4A shows that treatment of 25 μM and 50 μM of SF1126 decreased the baseline levels of phospho-ERK. Right panel of Figure 4A shows that administration of LY294002 (50 μM) and PD98059 (20 μM) significantly block phosphorylation of ERK. It is important to mention that pERK levels were significantly higher in non-treated (NT) cells grown in 10% FBS compared to the phospho-ERK level in non-treated (NT) cells grown in serum-free media (Figure 4A, Right panel).

In consistency with our results, Guha and coworkers reported a characteristic gain-of-function for EGFR signaling pathway in Ras-B8 astrocytomas (^12^ V-Ha-Ras transgenic mouse model). They showed an increased expression of wild type EGFR protein in the derivative Ras-B8 astrocytomas cell lines as well as in tumors in contrast to normal (WT) murine astrocytes or brains [6,8]. To gain further insight into the regulation of signaling pathways in V^12^-Ras-astrocytes derived from ^12^ V-Ha-Ras transgenic mice, we stimulated the glioma cells derived from these transgenic mice with EGF. Figure 4B shows that EGF (100 ng/ml) stimulation (under serum-free condition) increased the levels of phospho-ERK as compared to the non-stimulated control. Next, we examined the effect of SF1126 on EGF-mediated activation of ERK (phospho-ERK) in these cells. Figure 4B shows that the increase in the levels of phospho-ERK observed in the cells induced with EGF were significantly blocked in the presence of 25 μM and 50 μM of SF1126.

### Effect of SF1126 and LY294002 on the vitronectin-mediated migration in glioma cells from ^12^ V-Ha-Ras transgenic mice

**Figure 5:**
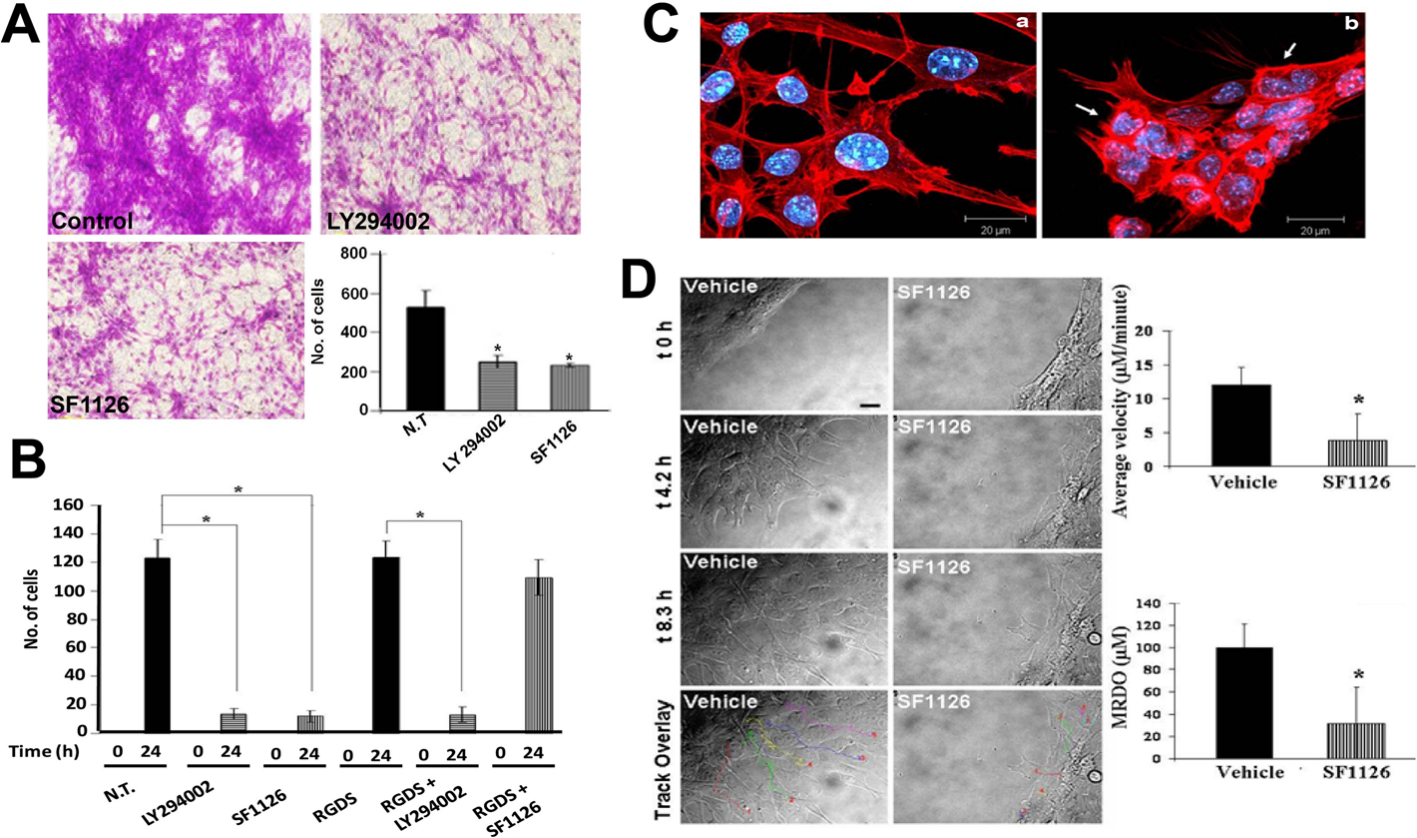
**Inhibition of vitronectin-directed migration of the glioma cells from 12V-Ha-Ras transgenic mice following the administration of SF1126 and its effect on the cortical distribution of filamentous actin. A**. Effect of SF1126 (50 mM) or LY294002 (50 mM) on vitronectin-directed migration of derivative glioma cells from 12V-Ha-Ras transgenic mice in transwell chambers. Bars represent the mean ± SD of number of cells migrated on vitronectin (in triplicates) in lower bottom panel. * P< 0.05. **B**. Effect of pre-pulse of RGDS peptide to the treatment of SF1126 and LY294002 on vitronectindirected migration of derivative glioma cells from 12V-Ha-Ras transgenic mice in scratch assays. Derivative glioma cells were allowed to migrate on vitronectin coated 24 well plates for 24 hours in presence or absence of either SF1126 (50 3 mM) or LY294002 (50 mM) following scratch on the confluent monolayer of the cells. In a separate experiment, cells were pulsed with RGDS peptide (50 mM) for 30 minutes before the administration of either SF1126 (50 mM) or LY294002 (50 mM). **C**. Effect of SF1126 (50 mM) on the cortical distribution of polymerized filamentous actin in the derivative glioma cells from 12V-Ha-Ras transgenic mice. **D**. Time-lapse confocal images of the effect of SF1126 on the vitronectindirected migration of the derivative glioma cells from 12V-Ha-Ras transgenic mice in scratch-wound assay. Vehicletreated (vehicle) and SF1126 treated (50 mM) cells were imaged separately for 8 hours (t 0 hour, t 4.2 hours, t 8.3 hours). Scale bar = 20 mm.The trajectory of the movement of the cells is characterized by two quantitative motility descriptors, average velocity (Upper bar diagram) and MRDO (maximum relative distance from the origin; Lower bar diagram) as shown in the bar diagrams. Bars represent Mean ± S.D. of the average velocity (mM/minute) and MRDO (mM) of the cells in presence of SF1126. *P<0.0025.

Migration is a characteristic property of the glioma cells [1]. Hall and his colleagues reported that RAS-mediated pathway plays an essential role in the movement of cells in chemotaxis and in wound-healing assays and attenuation of RAS signaling blocked the movement of cells [35]. The α_v_β_3_ integrin complexes are reported to be differentially expressed at the infiltrating peripheral margins of GBM [36] and are involved in the migration of cells during glioma-associated angiogenesis [37]. We therefore tested the effect of SF1126 on the vitronectin-mediated migration of glioma cells derived from the transgenic mouse in transwell migration chambers. Figure 5A shows that both SF1126 and LY294002 markedly reduced the migration of these cells on vitronectin.

We next investigated the effect of SF1126 on the vitronectin-mediated migration using wound healing scratch assay. Figure 5B shows that both SF1126 and LY294002 inhibited the migration of these cells for 24 hours on vitronectin. Since SF1126 is a RGDS-conjugated pro-drug, we wanted to test the specific effect of pre-pulse of RGDS peptide on the inhibitory effect of SF1126 on the vitronectin-mediated migration of cells. Pre-pulse of RGDS blocked the effect of SF1126, while it had no effect on the inhibitory effect of LY294002 (Figure 5B).

### Effect of SF1126 on the vitronectin-mediated distribution of filamentous actin in glioma cells from ^12^ V-Ha-Ras transgenic mice

Dynamic organization of actin cytoskeleton acts as the driving force for the cell movement [35]. In a migrating cell, the filamentous organization of the polymerized actin is required for its attachment to the extracellular matrix via integrin receptors [38]. The results described in Figure 5, clearly illustrate inhibition of vitronectin-mediated migration of SF1126 treated glioma cells derived from ^12^ V-Ha-Ras transgenic mice. These results prompted us to examine the effect of SF1126 on the distribution of polymerized cortical actin in these cells. Treatment of SF1126 for 30 minutes caused a significant change in the organization and distribution of cortical actin on vitronectin in the glioma cells (Figure 5C). The non-treated cells (photomicrograph a of Figure 5C) show an even distribution of cortical actin (polymerized) throughout the cell from center and the periphery in contrast to the treated cells (photomicrograph b of Figure 5C) where the cortical actin exhibits thickening around the periphery of the cells (arrow heads).

Since treatment of SF1126 inhibited the migration of glioma cells on vitronectin as well as showed an effect on organization and distribution of the cortical actin-filaments in the glioma cells, we were prompted to study real time movement of the live cells in vitro using time-lapse confocal video-microscopy. Figure 5D shows that the control cells derived from the V^12^ Ras transgenic animal exhibited directional motility into the scratch area with well-defined lamellae protruding from the leading edge of motile cells. The average distance traveled by control cells was ~100 μM over an 8.3-hour total time, and exhibited an average velocity of ~12.0 μM/minute. In contrast, the SF1126 treated cells exhibited poor or abnormal lamellae formation corresponding with limited migration, as the average distance traveled was ~45 μM over the same time with an average velocity of ~5.5 μM/minute. Treated cells that migrated also exhibited deviating paths relative to control cells, as shown in the track overlays, displaying the migratory paths traveled by the cells. Figure 5D shows that in presence of SF1126, both average velocity and MRDO of these glioma cells from ^12^ V-Ha-Ras transgenic mice decreased significantly compared to the vehicle treated cells.

### In vivo anti-tumor effect of SF1126 in mouse xenograft model

**Figure 6:**
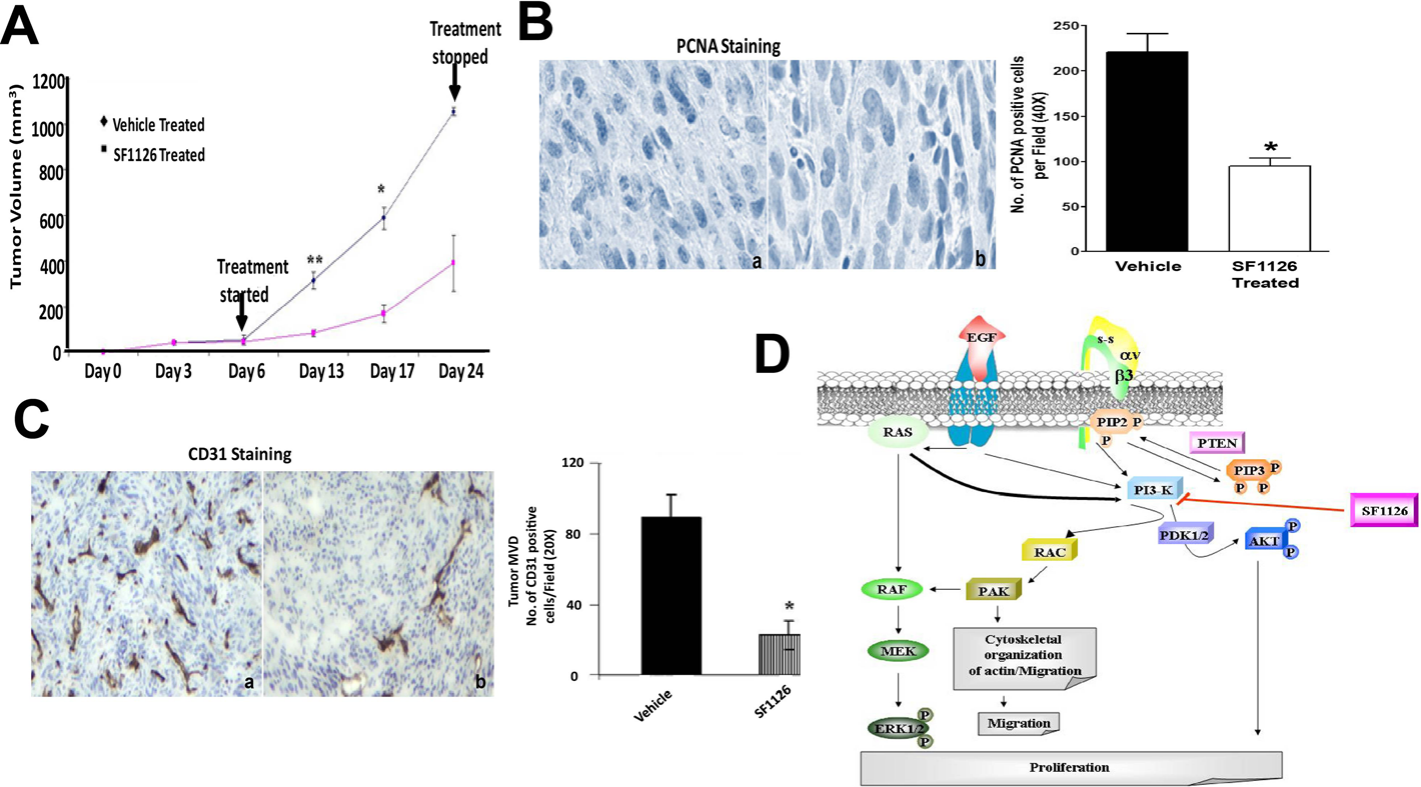
**Inhibition of in vivo growth of pre-established tumor in xenograft model following the administration of SF1126 and its effect on proliferation and angiogenesis. A**. Effect of SF1126 on the growth of the pre-established tumor in xenograft model. Athymic mice (n = 8-10) were subcutaneously implanted with the derivative glioma cells from ^12^ V-Ha-Ras transgenic mice (5 ×10 ^6^ cells in 100 μl PBS per animal). Mice bearing comparable volumes of tumor (80–100 mm^3^) were treated with 50 mg/kg/dose of either SF1126 or vehicle 3 times weekly (Monday, Wednesday and Friday) for 3 weeks. **B**. IHC staining of PCNA confirming that treatment of tumors with SF1126 block proliferation of tumors in the V12-Ras glioma cell xenograft model. Bars represent Mean ± S.E. of the number of PCNA positive tumor cells per randomly chosen field at 40× magnification. *P < 0.05. Data is representative of three independent experiments (n = 3). **C**. IHC CD31 staining confirming that treatment of tumors with SF1126 block expression of CD31 in the tumor endothelial cells from the pre-established subcutaneous tumors. Bars represent Mean ± S.E. of the number of CD31 positive tumor cells per randomly chosen field at 20× magnification. *P < 0.02. Data is representative of three independent experiments (n = 4). **D**. Schematic representation of the proposed model for the mode of anti-tumor action of novel PI3-kinase inhibitor, SF1126 in glioma. Mechanism of action of SF1126 in the regulation of PI3-kinase pathway and MAP-kinase pathway in the derivative glioma cells from ^12^ V-Ha-Ras transgenic mice is presented in the schematics.

The above mentioned results (Figures 1, 3, 4 and 5) clearly showed that SF1126 potently block phospho-AKT levels and inhibit the proliferation of the glioma cells derived from ^12^ V-Ha-Ras transgenic mice in vitro. These results prompted us to study the efficacy of SF1126 in vivo*.* The anti-tumor effects of SF1126 were tested in vivo using pre-established tumors (subcutaneous injection of the glioma cells from ^12^ V-Ha-Ras transgenic mice) in a mouse xenograft model. The animals bearing established tumors were injected (S.C.) with SF1126 50 mg/kg every alternate day (Monday/Wednesday/Friday) for 3 weeks. The results in Figure 6A demonstrate that SF1126 treatment significantly retarded the growth of the tumors in the athymic mice as compared to the vehicle treatment.

We next examined the expression of PCNA in the tumor tissues harvested from vehicle treated and SF1126 treated groups. A marked reduction in the expression of PCNA (Figure 6B) was observed in the tumors harvested from SF1126 treated mice compared to the vehicle treated animals. Since we observed a significant inhibition of tumor growth following the treatment of SF1126 in the xenograft model (Figure 6A), we studied the PCNA index and tumor microvessel density (MDV) in the tumors harvested from the animals treated with SF1126. Figure 6C shows that SF1126 significantly reduced PCNA index in the tumor confirming that treatment of tumors with SF1126 block proliferation of tumors in the V12-Ras glioma cell xenograft model. A quantitation of microvessel density (MVD) in control versus SF1126 treated tumors demonstrated a significant decrease in MVD in SF1126-treated tumors (Figure 6C) suggesting that pan-PI-3 K inhibition could also impair tumor growth through effects on tumor vasculature. Figure 6D shows the schematic representation of the proposed model for the mode of anti-tumor action of novel PI3-kinase inhibitor, SF1126 in glioma.

## Discussion

The rationale for selecting a PI-3kinase inhibitor as an effective therapeutic agent for the treatment of malignant gliomas is based on the fact that the PI-3kinase/AKT pathway is critical in the pathogenesis and progression of malignant gliomas [18,39,40]. This fact, along with the observed limited clinical efficacy of EGFR-inhibitors (an intact EGFR signaling-axis is required for an EGFR inhibitor to block PI3-kinase activity in glioma) suggest the necessity of PI3-kinase inhibitor for the effective treatment of the disease [41]. SF1126 is a vascular-targeted drug which showed considerable efficacy in B cell malignancies in Phase I clinical trials [42]. Recent reports from our lab have shown the efficacy of this drug in lymphoma and in various other xenograft models [22,23]. Herein, using ^12^ V-Ha-Ras transgenic mouse astrocytoma model we provide evidence that SF1126 can target both PI3-kinase and MAP kinase-ERK pathways in glioma cells by inhibiting proliferation and integrin-dependent migration of glioma cells.

The important observations of this study reveal: (i) a higher level of phospho-AKT in the glioma cells from ^12^ V-Ha-Ras mice compared to the primary astrocytes from wild type mice (Figure 3 inset), and (ii) an RGDS-dependent decrease in the levels of phospho-AKT in these cells following the treatment of SF1126 (Figure 3A and B). It is well established that in human GBMs, AKT is activated (in approximately 70% of the tumors) in association with the activation of receptor tyrosine kinases and/or the loss of PTEN [43]. Furthermore, it has been reported that the activation of the AKT pathway is sufficient to transform a anaplastic astrocytoma into glioblastoma multiforme [44]. The most probable reason for the high levels of pAKT observed in ^12^ V-Ha-Ras astrocytes can be attributed to the up regulated RAS signaling axis in these cells which might arise due to either ^12^ V-Ha-Ras transgene [7], and/or due to changes in the expression of EGF receptors as reported by Guha et al. [6]. Our model for the mechanism of action of SF1126 in glioma cells (Figure 6D) shows that PI3-kinase can be stimulated following (1) growth factor (EGFR) mediated signals, (2) up regulated RAS signals, and/or (3) integrin (α_v_β_3_) mediated signals, which increase the cellular levels of phospho-AKT. It is quite possible that SF1126 treatment inhibited the RAS-mediated up regulation of PI3-kinase signals in these cells. Because the state of activation of AKT plays an important role in the proliferation of tumor cells in GBM [19,43], this data encouraged us to test the effect of SF1126 on the growth of these cells in vitro and in vivo (Figure 3C and 6A).

We demonstrate that treatment of SF1126 decreased the baseline and EGF-stimulated levels of phospho-ERK in glioma cells derived from ^12^ V-Ha-Ras transgenic mice (Figure 4A and B). The PI3-kinase/AKT pathway has been reported to mediate the EGFR-dependent signals in GBM cells and the blockade of upregulated EGFR signals inhibited the activation of AKT [19]. Our model for the mechanism of action of SF1126 in glioma cells (Figure 6D) shows that ERK can be stimulated downstream of (i) growth factor (EGFR) mediated signals, (ii) upregulated RAS signals, and/or (iii) PI3-kinase mediated signals which activates MEK via RAF. Thus it is possible that an upstream activation of RAS activates PI3-kinase [20,32-34] which in turn upregulates MEK [45] leading to an increase in the levels of phospho-ERK. We propose that the treatment of SF1126 inhibited PI3-kinase-RAC-PAK-RAF mediated activation of phospho-ERK in these cells. Upregulation of RAS pathway is involved in the proliferation of tumor cells in human malignant astrocytomas [11]. Guha et al. reported that activation of both PI3-kinase (PI3-kinase/AKT) and MEK1/2 (MAP kinase -ERK1/2) pathways are required for the increase in proliferation of astrocytoma cells and growth of astrocytic tumors [10]. From the data, it is reasonable to suggest that the inhibitory effect of SF1126 on the proliferation (in vitro and in vivo) of these cells (Figure 3C and 6A) reflects the action of the drug on the levels of phospho-ERK and phospho-AKT.

Our results show a significant blockade of vitronectin-mediated migration in the glioma cells from ^12^ V-Ha-Ras transgenic mice in presence of SF1126 (Figure 5A). We interpret that the inhibition of migration of glioma cells on vitronectin following SF1126 treatment is in part due to its inhibitory effect on integrin-mediated activation of PI3-kinase pathway. AKT is an important downstream component of PI3-kinase mediated signals from the integrin receptors [46]. Studies by Joy et al. show that the migrating glioma cells preferentially exhibit high levels of phospho-AKT (at the migrating front). Our results also suggest that the treatment of SF1126 disrupts the cortical distribution of the polymerized actin in these cells (Figure 5C). Activation of PI3-kinase up regulates RAC1, a small GTP-ase that plays an important role in the cellular morphology and motility [47]. Upregulation of both RAS-MAP-kinase pathway and PI3-kinase pathway cause downstream activation of RAC1 [48]. We argue that the inhibitory effects of SF1126 observed on vitronectin-mediated migration in glioma cells may be mediated through its effects on both RAS-MAP-kinase and PI3-kinase pathways.

Therapeutic importance of the PI3-kinase/AKT pathway has been implicated in different brain tumors [33,40]. Assessment of multiple nodes of PI3-kinase pathway has been studied in the biopsy samples from GBM patients [18]. Recent report by Penas-Prado et al. indicated that targeted therapy can be an effective treatment option for the malignant gliomas [49]. Furthermore, an isoform specific inhibitor of PI3-kinase in the treatment of glioma has been suggested by Fan et al. [41]. Our results bring out an interesting feature of the mode of action of PI3-kinase inhibitors in glioma cells. We observed that in glioma cells (derivative glioma cells from ^12^ V-Ha-Ras transgenic mice), SF1126, a pan PI3-kinase inhibitor acts by attenuating both PI3-kinase and RAS-MAP kinase pathways. An understanding of the mechanism of action of novel agents like SF1126 in the regulation of the critical signaling pathways in gliomas at preclinical level will provide relevant information to design a targeted kinase inhibitor therapy based on the genetic and molecular signature of these tumors.

## Abbreviations

^12^ V-Ha-Ras astrocytoma: Astrocytoma cells from GFAP-^12^ V-Ha-Ras-transgenic mice
Derivative glioma cells: Glioma cells derived from GFAP-^12^ V-Ha-Ras-transgenic mice
EGF: Epidermal growth factor
EGFR: Epidermal growth factor receptor
GBM: Glioblastoma multiforme
MVD: Microvessel density
